# The canine vibrissal system as a highly innervated and functional sensory organ

**DOI:** 10.1038/s41598-025-91629-1

**Published:** 2025-03-17

**Authors:** Dorothea Döring, Helen E. Müller, Sophie Franzmeier, Kaspar Matiasek, Andreas Blutke, Sven Reese

**Affiliations:** 1 Chair of Animal Welfare, Ethology, Animal Hygiene and Husbandry, Department of Veterinary Sciences, Ludwig-Maximilians-Universität München, Munich, Germany; 2 Institute of Veterinary Pathology, Ludwig-Maximilians-Universität München, Munich, Germany; 3 Chair of Anatomy, Histology and Embryology, Department of Veterinary Sciences, Ludwig-Maximilians-Universität München, Munich, Germany

**Keywords:** Vibrissae, Dogs, Histology, Sinus hairs, Tactile hairs, Microvibrissae, Zoology, Anatomy

## Abstract

Little is known about the importance of the vibrissae in domestic dogs and no studies have been published to date. The shaving of the vibrissae is still common in dogs, particularly in preparation for dog shows. In this publication, we show how dogs use their vibrissae with 17 video clips from 11 individuals. We also performed histological, transmission electron microscopic and immunohistochemical analyses to characterise the histomorphology of canine follicle-sinus complex (FSC). Tissue samples were taken from six dog carcasses. The videos show that dogs respond to mechanical stimulation of their vibrissae. Touching the superciliary and mystacial vibrissae induces a reflex blinking of the eyelids. Dogs also move their mystacial vibrissae forward to explore objects. Histomorphological and ultrastructural analyses confirmed that canine mystacial FSCs have the typical general histomorphology of mammalian sinusoidal vibrissae with dense innervation and specific mechanoreceptor structures. The follicles of the small hairs on the upper lip also have the typical canine FSC structure and, therefore, can be identified as ‘microvibrissae’. In conclusion, there is sufficient evidence to suggest that the vibrissae of the domestic dog are a functional sensory organ and should not be shaved for cosmetic reasons to protect the physical integrity of the dog.

**Supplementary Information:**

The online version contains supplementary material available at 10.1038/s41598-025-91629-1.

## Introduction

Sinus hairs or vibrissae, called *pili tactiles* in Latin^1^, are found in domestic dogs on the upper and lower lip, under the throat, on the cheeks (as a double tuft) and above the eyes^2,3^. Each vibrissa is deep-seated within a follicle in the skin. This follicle is anatomically different from other hair follicles, containing blood-filled compartments and dense innervation. It is therefore referred to as the vibrissal “follicle-sinus complex” (FSC) and is considered a sensory receptor of the mammalian integumentary system^4^.

According to veterinary histology textbooks, the FSC of the domestic dog is of the sinusoidal type^5^. More than 80 years ago, Ueda^6^ measured the sinus hairs and follicles of the domestic dog, presented them as a histological section and compared them with those of the Japanese cat. The sinus hair follicles of dogs and cats were found to be quite similar. Studies by Muchlinski et al.^7^ showed that the mystacial vibrissae in domestic dogs, as in domestic cats, belong to the ordered vibrissae with intrinsic musculature. Ramírez et al.^8,9^ demonstrated Merkel cells (mechanoreceptors) in the canine FSC.

Vibrissae are common in mammals^10,11^, with only a few species, such as humans, lacking FSCs. However, studies on the function of vibrissae are only available for a few species, mainly laboratory rats and mice and some species of pinnipeds, marsupials and sirenians (see review by Grant and Goss^11^). There are also some publications on cats. As early as 200 years ago, Broughton^12^ closed the eyes of a kitten completely, cut off the vibrissae and found that it collided with objects as it moved. About 100 years later, Schmidberger^13^ performed experiments on cats that had been surgically blinded and had their vibrissae removed. The blind cats with vibrissae moved upright, quickly and confidently through a room full of obstacles, resembling sighted animals in their movements. In contrast, the blind cats without vibrissae either did not move at all or moved very cautiously, slowly and in a crawling posture, feeling their surroundings with their lips and nose or bumping into obstacles. The studies by Crémieux et al.^14^ confirmed that cats without vibrissae move much slower through a maze in the dark than cats with vibrissae. Gogan et al.^15^ showed that stimulation of the cat’s mystical vibrissae resulted in withdrawal and rotation of the eyeball on the same side of the face.

To date, there are no studies investigating the function of the vibrissae in dogs. Some anecdotal descriptions can be found in the literature. For example, Coren^16^ reported a case of a blind collie who, after his vibrissae were amputated, became disoriented, bumped into objects and missed the water bowl with his snout. McGill^17^ described two cases of Viszla dogs working in the field with injuries to the face/eyes after their vibrissae had been clipped. Van Horn^18^ described reflex blinking when the mystacial vibrissae were touched, Döring et al.^19,20^ described reflex blinking when the superciliary vibrissae were touched. According to Beaver^21^, the movement of the dog’s vibrissae contributes to facial expression: They are raised during aggressive behaviour and lowered during submissive behaviour. It is therefore suggested that canine vibrissae also have a function in intraspecific communication.

Unlike cat vibrissae, dog vibrissae are often not considered worthy of preservation. It has been common practice for decades to cut off the vibrissae at the groomer, especially in preparation for dog shows, for purely aesthetic reasons^17,22,23^. Muzzle shaving is particularly common in poodles. The cover of the FCI (Federation Cynologique Internationale) poodle (caniche) breed standard shows a poodle with a shaved muzzle^24^. In his expert opinion, Dehnhardt^25^ stated that the vibrissae of dogs are usually reduced in number and structure, to justify the shaving of poodles’ muzzles. However, he did not provide any specific scientific evidence to support this claim.

To date, the histomorphology of the canine FSC and its innervation have not been extensively studied. Furthermore, it has not been proven whether the small hairs on the upper lip (under the nose) are actually functional “microvibrissae” or not. In the present work, we show how dogs use their vibrissae for exploratory and feeding behaviour and how their vibrissae have a protective function. We also provide histomorphological and ultrastructural evidence that canine vibrissae are highly innervated sensory organs.

## Materials and methods

### Behaviour

#### Animals

Veterinary colleagues and students of the Veterinary Faculty, LMU Munich, were asked to observe and film their dogs in certain situations to demonstrate how dogs use their vibrissae: Some owners touched the mystacial vibrissae of their dogs while they were sleeping. Other owners touched the vibrissae when their dogs were awake but calm and relaxed. Some owners filmed their animals exploring objects or eating food. In this way, the Chair of Animal Welfare, Ethology, Animal Hygiene and Husbandry collected a number of videos of different individuals in diverse situations. A selection of the most illustrative videos were chosen as examples for this publication (Table 1).

These 17 video recordings of 11 privately owned dogs were made by their owners in their familiar environment using their own smartphone cameras. To ensure that the dogs were relaxed, they remained in their familiar surroundings and were touched only by their owners. For animal welfare reasons, it was intended that the dogs were relaxed and not stressed. The videos do not represent animal experiments, but rather simply observations of the behaviour of privately kept dogs in usual everyday situations. It was therefore deliberate that the videos were not recorded in a standardised situation or environment and that the dogs were not touched by strangers.

**Table 1:** Overview of the privately owned dogs that were filmed by their owners. “sm” = slow motion, “f” = female, “m” = male, “vet” = veterinarian.

Breed	Sex	Owner (video recording)	Action of owner	Reaction of dog	No. of video
Mixed breed	f	Vet	Touching mystacial vibrissae while sleeping	Awakes	1
Mixed breed (Poodle with Border collie)	m	Cousin of vet student	Touching moustache while sleeping	Awakes	2
Miniature poodle	m	Friend of vet student	Touching moustache while sleeping	Awakes	3
Labradoodle (Labrador retriever with Poodle)	f	Vet student	Touching mystacial vibrissae while sleeping	Awakes, eye blinking	4
Goldendoodle (Golden retriever with Poodle)	m	Vet student	Touching mystacial vibrissae while awake	Eye blinking	5
Australian Shepherd	f	Vet	Touching mystacial vibrissae while awake	Eye blinking	6
German longhaired pointer	m	Vet	Touching mystacial vibrissae while awake	Eye blinking	7
King poodle	f	Vet	Touching mystacial vibrissae while awake	Eye blinking	8, 9
Mixed breed	f	Vet	Touching superciliary vibrissae while awake	Eye blinking	10,11
German longhaired pointer	m	Vet	Touching superciliary vibrissae while awake	Eye blinking	12
Australian Shepherd	f	Vet	Touching superciliary vibrissae while awake	Eye blinking	13
Mixed breed	f	Vet student	Squeak with a squeaky toy under a blanket	Movement of the mystacial vibrissae forward	14
Mixed breed	m	Vet student	Showing a hairbrush	Movement of the mystacial vibrissae forward, touching with the vibrissae of the left side of the muzzle	15 (sm)
Mixed breed	m	Vet student	Lay out a track of treats on the floor	Touches the treats with the small vibrissae of the upper lip before eating	16 (sm)
Mixed breed	m	Vet student	Scatter treats on gravel floor	“Hovering” with the snout above the ground, touching the pebbles and treats with the small vibrissae of the upper lip	17 (sm)

Video sources: A. Schwarzer (video 1), S. Prause (video 2), L. Peters (video 3), S. Stützle (videos 4, 5), S. Bergmann (videos 6, 13), J. Heck (videos 7, 12), A. Bartels (videos 8, 9), A. Grott (videos 10, 11), J. Dirscherl (video 14), H. Kunisch (videos 15–17).

### Histology

#### Histological preparations of the different vibrissae sites

The facial skin of a dead male Labrador Retriever puppy, 7 weeks old, submitted for necropsy to the Institute of Veterinary Pathology, LMU Munich, was used. Follicles of the mystacial and supraorbital sinus hairs and some of the small hairs of the upper lip near the oral mucosa were removed, fixed in 4% neutral buffered formalin solution, embedded in paraffin and sections of 3 μm were stained with HE (haematoxylin-eosin)^26^.

#### Histomorphological and ultrastructural analyses of canine mystacial FSCs

Histological and transmission electron microscopic (TEM) analyses were performed on tissue samples of mystacial vibrissae excised from the cadavers of five adult dogs of different mixed breeds of both sexes submitted for routine necropsy at the Institute of Veterinary Pathology, LMU Munich. For histological analysis, tissue samples were fixed in 4% neutral buffered formalin solution for < 12 h, trimmed and routinely embedded in paraffin or plastic resin (GM/MMA or Epon) as previously described^27,28^. Sections with a nominal thickness of 3 μm (paraffin sections) and 1.5 μm (GMA/MMA sections) were stained with HE. For TEM, 1 mm³ FSC samples were fixed in 2.5% neutral buffered glutaraldehyde solution, post-fixed in 1% osmium tetroxide and routinely embedded in Epon resin. Semithin sections (0.5 μm) were stained with toluidine blue/safranin O. Toluidine blue staining is particularly suitable for light microscopic visualization of nerves in Epon resin embedded tissue^29^. Ultrathin sections of 70–80 nm thickness were stained with uranyl acetate and lead citrate and examined on an EM900 electron microscope (Zeiss, Eching, Germany) as previously described^28^.

#### Immunohistochemistry

To demonstrate dense FSC innervation, PGP 9.5 (protein gene product 9.5), a universal neuronal marker (cytoplasmic enzyme), was detected by immunohistochemistry in paraffin sections using a polyclonal rabbit anti-PGP 9.5 primary antibody (Code No. Z5446, Dako Cytomation, Denmark, dilution 1:100). Biotinylated goat anti-rabbit IgG antibody (BA-1000, Vector, Peterborough, UK, dilution 1:200) was used as secondary antibody. Immunoreactivity was visualised using the Elite ABC Kit Peroxidase (HRP) (Cat. PK-6100, Vectastain Laboratories, CA, USA) with 3,3-diaminobenzidine tetrahydrochloride dihydrate (DAB) as chromogen and hemalaun as nuclear counterstain. FSC sections stained with buffer instead of primary antibody were used as negative controls.

## Results

### Behaviour

The dogs in videos 1–4 woke up when their mystacial vibrissae or moustaches were touched. In videos 2 and 3, the sleeping dogs had previously been touched on their fur but did not react.

In videos 5–9, the dogs blinked their eyes when the mystacial vibrissae were touched.

The dogs also blinked when the superciliary vibrissae were touched, as can be seen in videos 10–13.

Video 14 shows movements of the mystacial vibrissae while the owner squeaks a squeaky toy first under and then over a blanket.

Video 15 shows a dog spreading its mystacial vibrissae forward to examine a hairbrush. The video is in slow motion.

The owner laid a trail of treats on the floor and filmed the dog eating the treats in slow motion (video 16). You can see how the dog slides his snout over the floor and touches the treat with the small vibrissae on his upper lip, just before he takes it with his tongue and lips.

In video 17, the owner scattered treats between pebbles and filmed the dog in slow motion as he searched for and ate the treats. The dog can be seen hovering over the pebbles with its snout at a short distance from the ground, alternating between sniffing with its nose and touching with the microvibrissae of its upper lip before picking up the treat.

**Fig. 1:**
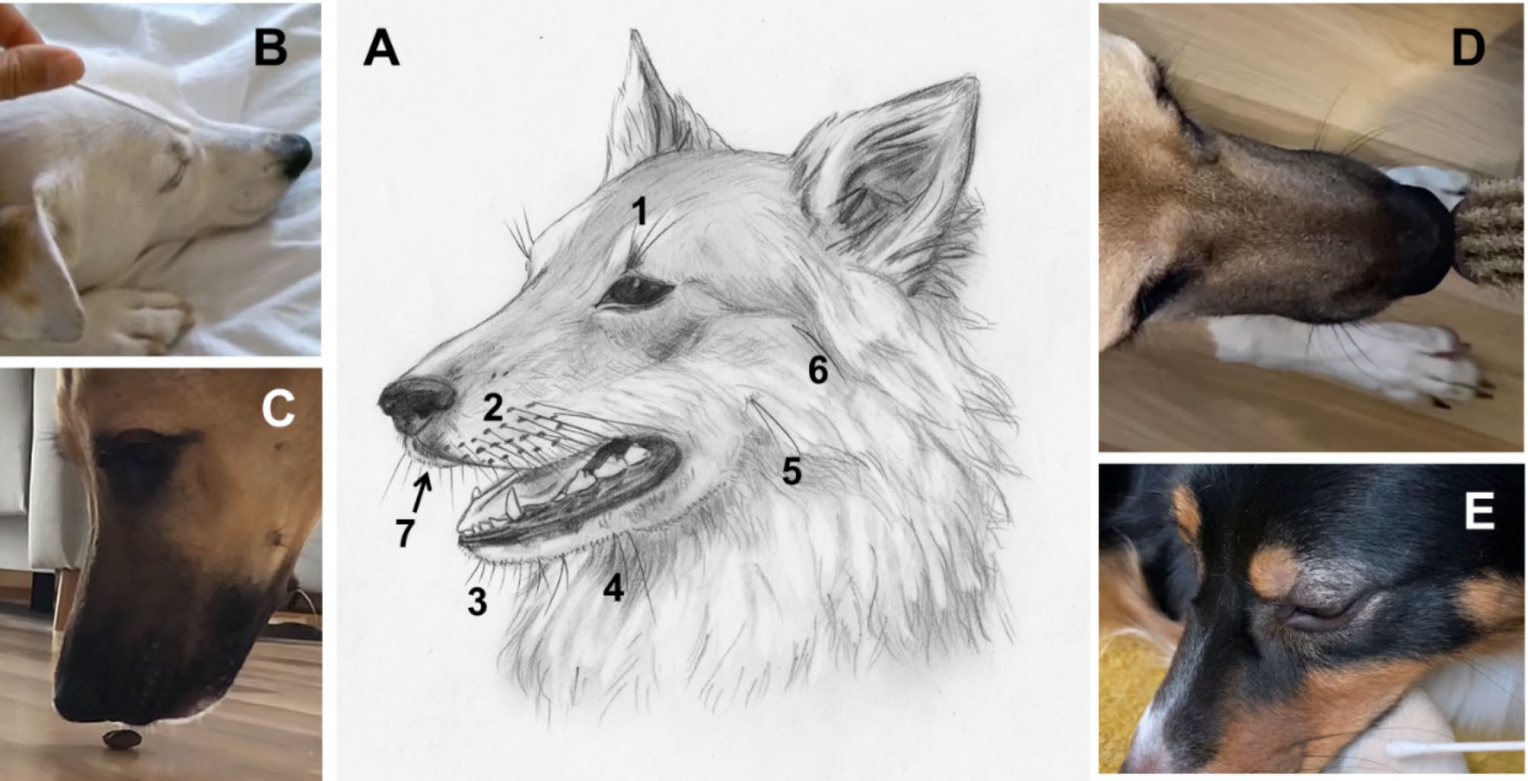
Vibrissae and behaviour. (**A**) Drawing showing the different locations of the domestic dog’s vibrissae: Pili tactiles supraorbitales (superciliary vibrissae, 1), pili tactiles labiales superiores (mystacial vibrissae, 2), pili tactiles labiales inferiores (3), pili tactiles mentales (4), pili tactiles buccales (5), pili tactiles zygomatici (6), labial microvibrissae (7) (drawing: Dorothea Döring). (**B**) Eye blinking when owner touches the superciliary vibrissae (video 11). (**C**) Mixed breed touches the treats with the small vibrissae (microvibrissae) of the upper lip before eating (video 16). (**D**) Mixed breed moves mystacial vibrissae forward to explore a hair brush (video 15). (**E**). Australian Shepherd blinkes when owner touches mystacial vibrissae (video 6).

Figure 1 shows an overview of the behavioural patterns associated with the vibrissae at three locations.

### Histological preparations of the different vibrissae sites

**Fig. 2:**
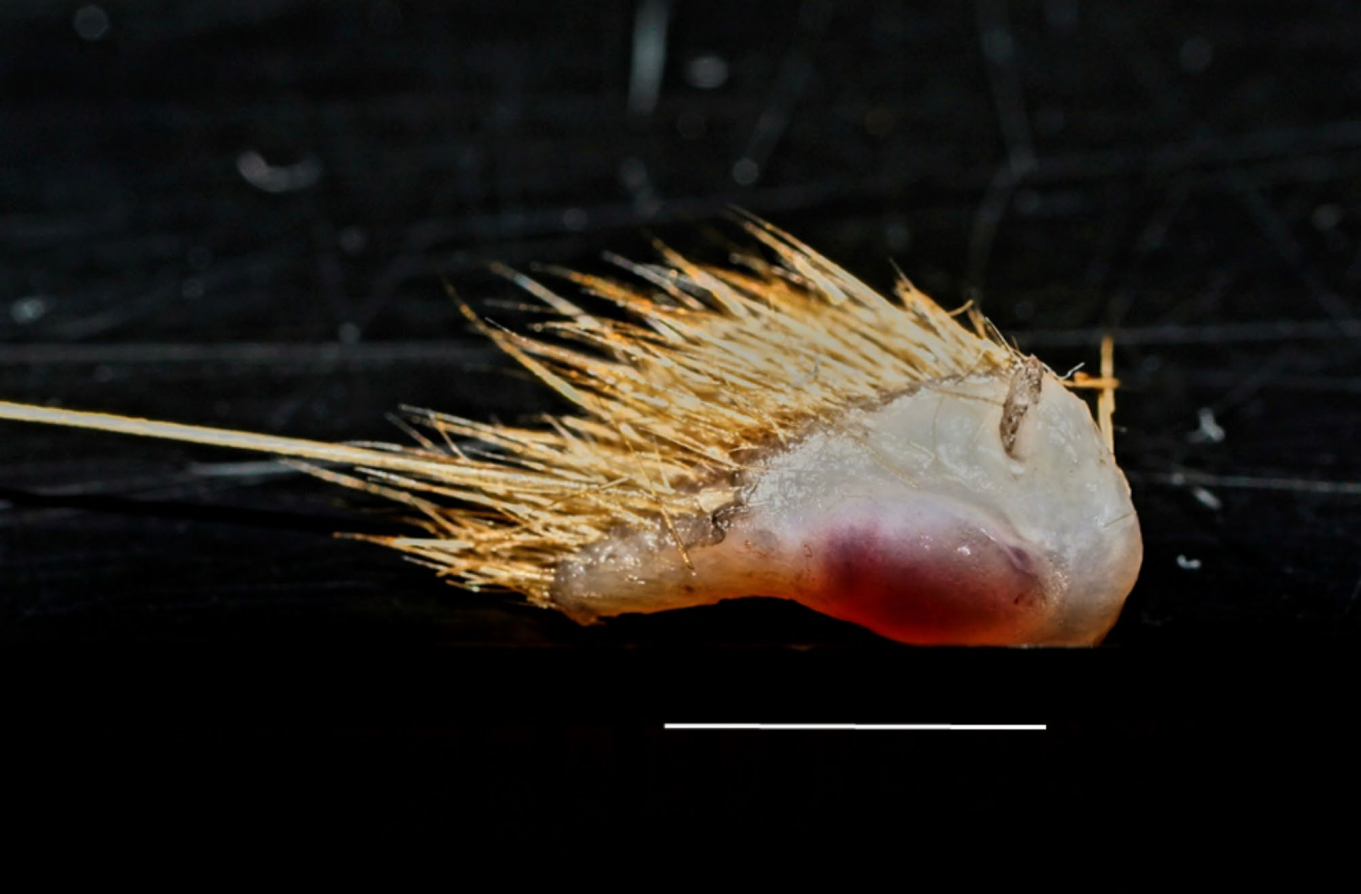
An excised piece of upper lip skin with mystacial sinus hair. One can see with the naked eye the follicle surrounded by blood sinuses, about the size of a small grain of rice (Labrador-Retriever puppy, 7 weeks of age). Scale-bar = 5 mm.

Figure 2 shows a mystacial sinus hair in a piece of skin excised from the upper lip: macroscopically, the follicle is seen to be surrounded by blood sinuses.

**Fig. 3:**
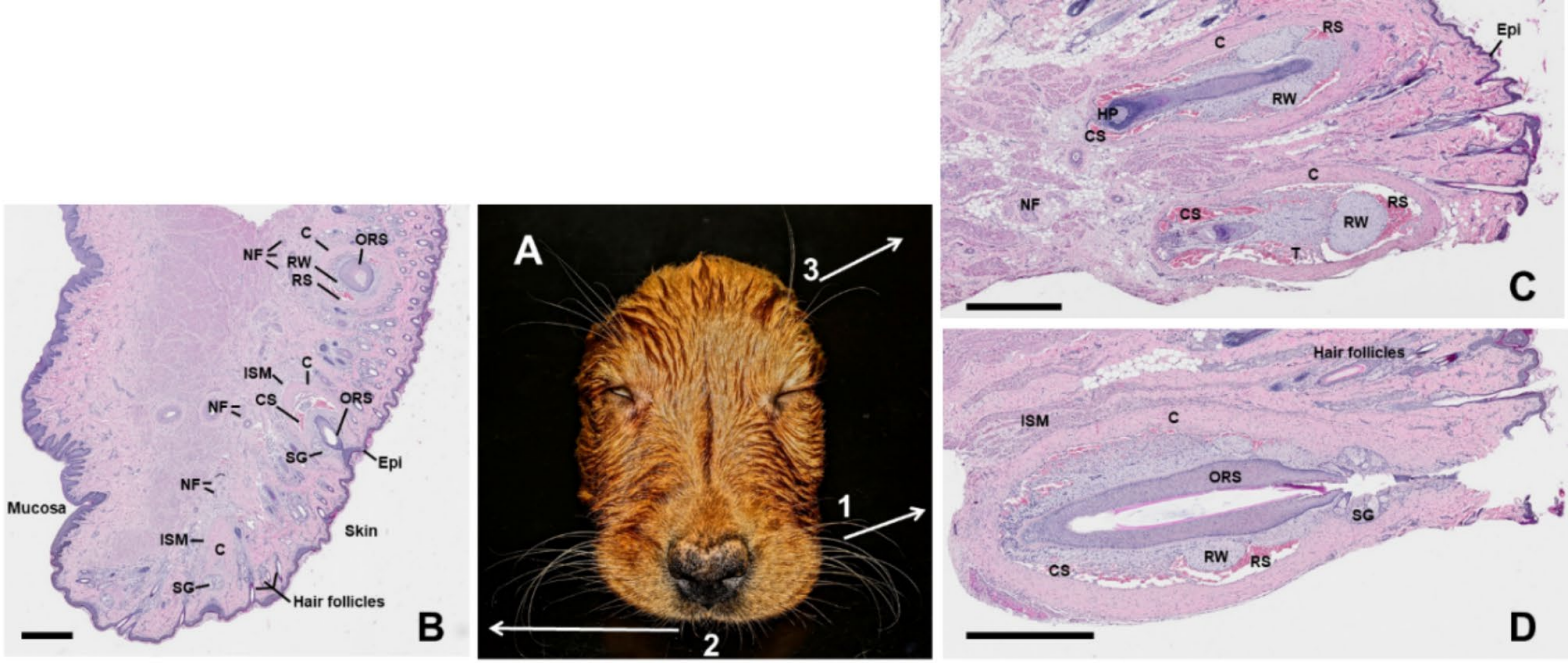
Histology of follicle sinus complexes (FSC) in three locations in the dog‘s face. (**A**) Facial skin of a dead Labrador Retriever puppy with mystacial vibrissae (1), labial microvibrissae (2) and superciliary vibrissae (3). B-D. Histological images of labial (**B**), superciliary (**C**) and mystacial (**D**) FSC. Image B shows a sagittal section of the upper lip with transition from mucosa area to skin area. Three FSC of microvibrissae are labeled, they are cut on different levels. Paraffin sections, HE staining. Scale-bars = 1 mm. *RS = proximal circumferential ring sinus*,* RW = ringwulst*,* CS = distal cavernous sinus*,* T = meshwork of trabeculae*,* C = connective tissue sheath*,* NF = nerve fibers*,* ORS = outer root sheath*,* Epi = epidermis*,* HP = hair papilla*,* ISM = Intrinsic striated muscles*,* SG = sebaceous gland.*

Figure 3 shows the arrangement of the mystacial, superciliary and small vibrissae of the upper lip of a Labrador puppy. In the histological sections (HE staining, Fig. 3C and D), the typical structure of the FSC of the domestic dog is demonstrated in the mystacial and superciliary follicles. The typical structure of the FSC can also be seen in the small hairs on the upper lip below the nose close to the mucocutaneous junction (Fig. 3B). Similar to the FSC of the large vibrissae, the follicles of the small vibrissae are surrounded by intrinsic striated muscles and there are thick bundles of nerve fibres nearby (Fig. 3B).

### Histomorphology and ultrastructure of canine mystacial FSC

**Fig. 4:**
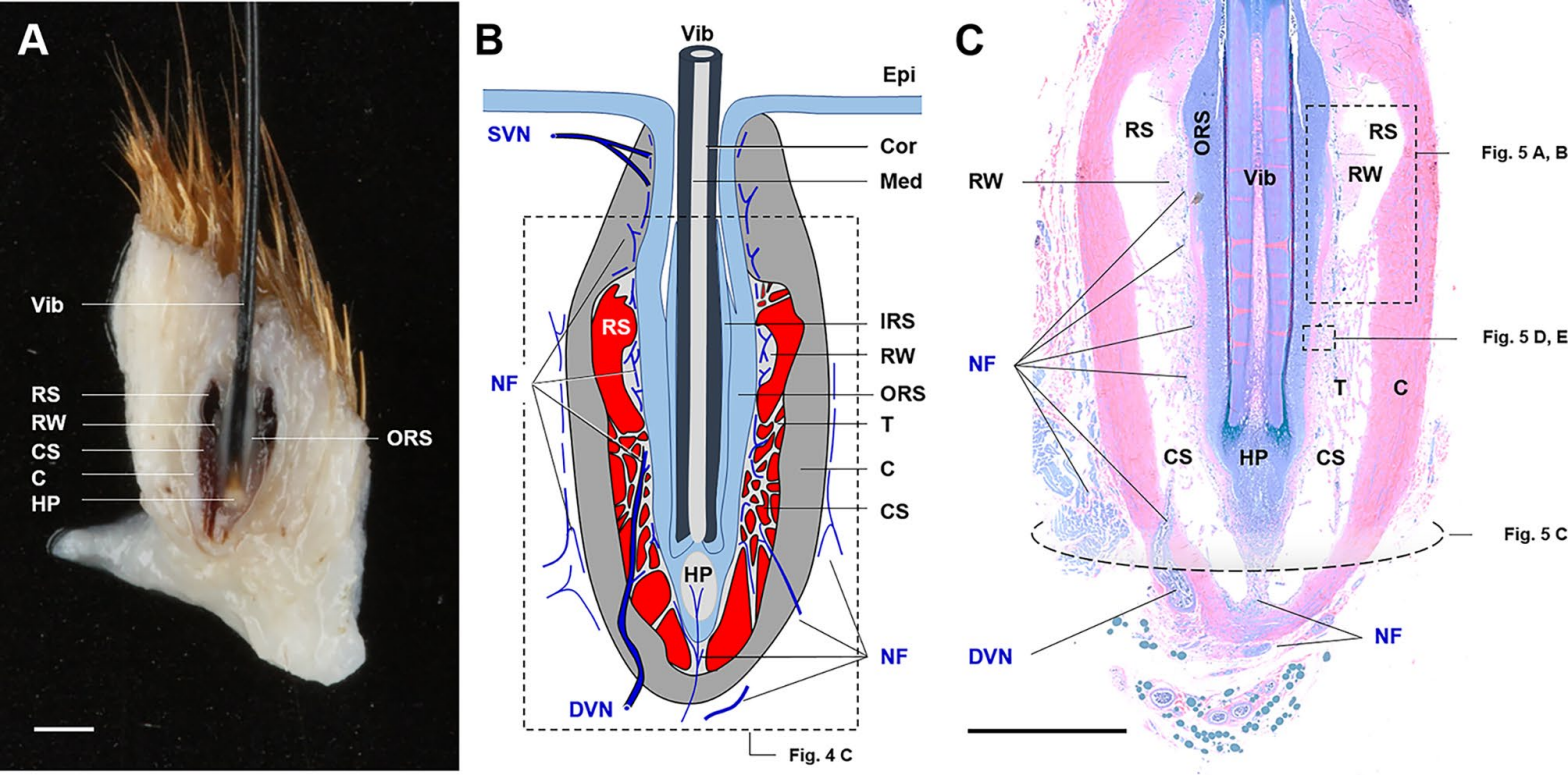
Morphology of canine mystacial follicle-sinus complex (FSC). (**A**) Mid-saggital section of a mystacial FSC. Formalin-fixed tissue. Scale-bar = 1 mm. (**B**) Schematic illustration of the FSC-histomorphology. (**C**) FSC-histology corresponding to the section plane orientation in A and B. Toluidine blue and Safranin O stained semithin section of Epon-embedded tissue. Scale-bar = 1 mm. FSC consist of a hair follicle with a vibrissa (Vib), surrounded by a proximal circumferential ring sinus (RS) with a prominent ringwulst (RW), as well as a continous distal cavernous sinus (CS) with a meshwork of trabeculae (T) and are encapsulated by a thick connective tissue sheath (C). FSC are densly innervated by nerve fibers (NF) that penetrate the capsule and branch in the trabeculae, the ringwulst and along the outer root sheath (ORS) of the follicle. Epi = epidermis, IRS = inner root sheath, SVN = superficial vibrissal nerve, DVN = deep vibrissal nerve, Cor = cortex, Med = medulla, HP = hair papilla. Tissue samples were taken from the carcasses of five adult dogs of different mixed breeds of both sexes submitted for routine necropsy at the Institute of Veterinary Pathology, LMU Munich.

**Fig. 5:**
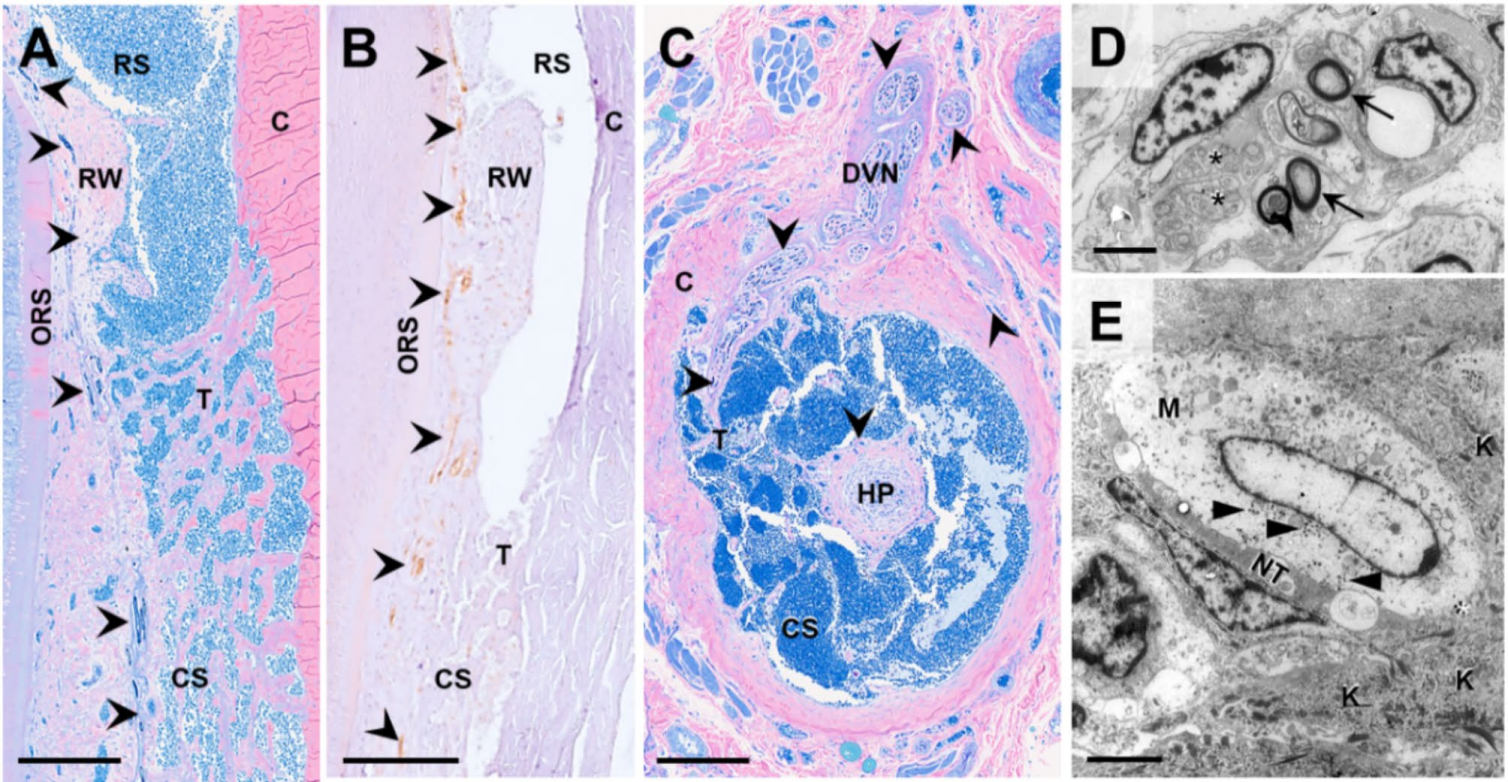
Histomorphology and ultrastructure of canine mystacial follicle sinus complex (FSC). (**A**,**B**) Mid-saggital section of a mystacial FSC at the level of the ring sinus (RS) and the cavernous sinus (CS). Scale-bar = 1 mm. (**A**) Toluidine blue and Safranin O stained semithin Epon-section. (**B**). Immunohistochemical detection (brown reaction product) of the pan-neuronal marker PGP9.5 in a paraffin section (chromogen: DAB, nuclear counterstaining: hemalaun). (**C**) FSC cross section at the level of the hair papilla (HP). Arrowheads indicate nerve fibers penetrating the capsule (**C**) and branching in the trabeculae (T), the ringwulst (RW) and along the outer root sheath (ORS) of the follicle. DVN = deep vibrissal nerve. (**D**,**E**) Ultrastructure (transmission electron microscopy) of a vibrissal nerve fiber bundle (**D**) with myelinated (arrows) and non-myelinated (black asterisks) nerve fibers in a trabecula of the cavernous sinus and a Merkel nerve ending (mechanoreceptor) attached to the ORS (**E**). The Merkel nerve ending comprises a Merkel cell (M) containing numerous characteristic osmiophilic granules (arrowheads) and cytoplasmic processes (white asterisk) extending between the adjacent keratinocytes (K) in the ORS and a discoid nerve terminal (NT) with abundant mitochondria. Scale-bar = 2 μm.

The canine mystacial vibrissae examined in this preliminary study exhibited the previously described general histoarchitecture of a mammalian vibrissa of the sinusoidal type^6,30,31^ with a ring and a cavernous sinus, a prominent ringwulst adjacent to the outer root sheath of the follicle, dense innervation with nerve fibres, and a peripheral thick connective tissue capsule (Fig. 4). Figure 5 shows the histomorphology and ultrastructure of the FSC with its dense innervation (Fig. 5A–C). Transmission electron microscopy revealed a specific type of mechanoreceptor (Merkel nerve endings) at the outer root sheath/sinus interface in various segments of the follicle, and abundant nerve fibre bundles with myelinated and non-myelinated fibres in the sinus trabeculae and FSC capsule (Fig. 5D, E).

The representation in the figures is exemplary. Additional tissue samples and analyses from other individual dogs are available at the Institute of Veterinary Pathology and the Chair of Anatomy, Histology and Embryology, LMU Munich.

## Discussion

Our video clips provide insight into how dogs use their vibrissae.

Videos 1–13 show that all dogs reacted to touching their vibrissae. This proves that these nine dogs of different breeds and both sexes have functioning vibrissae with which the dogs can sense touch. Even though the videos show unstandardised situations with different floors, with or without background noise, and the dogs were touched with different objects (pens or cotton swabs), all of these videos show that the dogs reacted to touch. The dogs in videos 5–13 blinked their eyes when the superciliary or mystacial vibrissae were touched, as described by Van Horn^18^, Coren^23^, and Döring et al.^19^. This is a protective reflex to protect the eyes and face, for example when running through thickets. Unlike humans, who can feel their way with their hands, dogs typically lead with their heads, making this a valuable protective mechanism. In a recent study of blind dogs^32^, some mystacial vibrissae were artificially lengthened by gluing pig bristles to them. The dogs used their vibrissae to navigate a maze and were able to maintain a greater distance from the maze walls with the help of these artificial extensions. The results of this study confirm that the dogs’ FSCs were functional and that their sensory information was used by the dogs.

Since dogs are unable to visually perceive the area under their snout due to anatomical conditions such as eye placement, snout shape, and hyperopia^33^, the vibrissae, including the microvibrissae on the lips, are used for touching and thus for exploratory behaviour, feeding behaviour, and orientation at close range. Video 16 shows that the dog touches the treats with his microvibrissae before picking them up. Perhaps dogs, like rats, are able to detect the texture of objects with their microvibrissae. This would explain why the dog in video 16 was able to accurately pick up small pieces of treats between the pebbles. Our videos 14 and 15 also show the rostral movement of the vibrissae (also described by Coren^23^) to actively explore specific objects, presumably using the intrinsic striated muscles of the FSC.

These other functions of the vibrissae were suggested: When following scent trails, dogs move their snout close to the ground (as can be seen in video 17), even when moving quickly. It is possible that the vibrissae are used to maintain an appropriate distance from the ground and to detect unevenness. This may protect the dog from injury to the lips and muzzle.

Dogs, as scent orientated hunters, appear to benefit from being able to use their vibrissae to determine wind direction^17^. This may enable them to determine the direction of odour source. Experiments on rats have shown that the vibrissae play a role in detecting wind direction: after the vibrissae were amputated, the animals were less able to locate the direction of an air draught than before, when the vibrissae were intact^34^. Although there are no studies in domestic dogs, it seems plausible that the vibrissae may play a role in the localisation of air draughts and transported odours.

Our videos are only examples of demonstrations on privately owned dogs. All dogs responded in this way, regardless of the environmental conditions. However, further research would be necessary to prove that the observations made here can be extrapolated to every dog and every situation.

In contrast to several other species, such as rodents^29,35^, cats^36^, marine mammals^37^ and exotic species^31^, the morphology and innervation of the canine FCS have not been analysed in detail. In the present work, it was confirmed that canine mystacial FSCs have the typical general histomorphology of mammalian vibrissae, with dense innervation and specific mechanoreceptor structures. This demonstrates that the canine FSC is a functional, highly innervated sensory organ.

We are currently considering further studies to provide a detailed, advanced characterisation of the types, distribution and densities of vibrissal mechanoreceptors and innervation of canine FSCs in different breeds, including poodles.

The results of the histological analyses also show that the small hairs on the upper lip near the junction with the oral mucosa also have the typical structure of sinus hair follicles, so they are obviously “microvibrissae”, probably similar to the microvibrissae in rats as described by Grant et al.^38^ and Kuruppath et al.^39^. In rats, the microvibrissae are used to palpate objects^38^ and allow the animals to discriminate between different surfaces based on their texture and shape^39^. The presence of a thick layer of striated muscle surrounding canine microvibrissal FSCs was demonstrated by histological analysis (Fig. 3B). Unlike smooth muscle, striated muscle allows voluntary movements. Therefore, these striated muscles allow the dog to perform an active, voluntary movement of the vibrissae to explore surfaces. However, involuntary piloerection is caused by smooth muscle contraction (arrector pili muscle^40^). The type of muscle therefore represents a significant anatomical difference between vibrissae and body hairs. Thus, for the first time in the dog, we were able to demonstrate the existence of microvibrissae, not yet listed in the Nomina Anatomica Veterinaria^1^ for any domestic species, and their active use by the dog when inspecting treats lying on the ground.

As it has been common practice for many years to shave the vibrissae of dogs, McGill spoke out against this as early as 1980^22^ and 1982^17^, stating: “(.) vibrissae are sense organs of potential major significance to the dogs (.)”^17^. So far, it has not been established to protect the dog’s vibrissae. Poodle breeders and owners continue to shave the muzzles of their poodles, claiming that this is necessary for dog shows. However, the FCI requires a moustache for the Lion Clip and the Modern Clip, thus protecting the mystacial vibrissae: “A moustache is required for all subjects”. For the English Clip, “(.) the moustache is optional”^24^. We also require protection of the vibrissae in other areas of the dog’s head.

As the hair on the poodle’s face is constantly growing back and can interfere with vision, the hair on the poodle’s face must be trimmed to a certain extent. However, shaving the snout is not necessary and should be considered to impact negatively on the dog’s sensory functions. Clipping the vibrissae, therefore, should be prohibited from an animal welfare perspective.

## Conclusion and recommendations

There is no doubt that the vibrissae of domestic dogs have intact, highly innervated follicles. Dogs respond reproducibly to the touch of their vibrissae; they blink when the superciliary and mystacial vibrissae are touched. They also use their microvibrissae to sense beneath their snout.

This is the first publication to provide a detailed histomorphological and ultrastructural analysis of the canine FSC and the first description of microvibrissae in the domestic dog. The small hairs on the upper lip not only have the same typical structure as the canine FSC, but are also surrounded by a thick layer of striated (“intrinsic”) muscle that allows voluntary movements.

These facts show that shaving the vibrissae is not acceptable for animal welfare reasons, as it renders a sensory organ temporarily unusable. The vibrissae of domestic dogs should not be cut or shaved in any breed. Dogs with curly hair that grows back, such as poodles, should have a well-groomed beard.

The present study is a pilot study and exemplary presentation on a small number of dogs. Future studies should investigate whether there are breed differences, particularly with regard to innervation and functionality of the FSC. In future dog breeding, attention should be paid to a fully developed vibrissal system.

## Electronic supplementary material

Below is the link to the electronic supplementary material.

Supplementary Material 1

## Data Availability

All data generated or analysed during this study are included in this published article.
