# Supplementary material for: The canine vibrissal system as a highly innervated and functional sensory organ
Source: Sci Rep. 2025 Mar 17;15:9212. doi: 10.1038/s41598-025-91629-1 (PMC11914073; doi:10.1038/s41598-025-91629-1)
Supplement: Supplementary file 1 — Supplementary Material 1 [file 41598_2025_91629_MOESM1_ESM.docx]

**Videos**

The videos can be accessed at:

<https://cast.itunes.uni-muenchen.de/vod/playlists/XS9zG57bHW.html>
